# Adult deterioration detection system (q-adds) based rapid response system (rrs) reduces severity of illness and length of stay of icu admissions from the ward in a regional hospital

**DOI:** 10.1186/2197-425X-3-S1-A141

**Published:** 2015-10-01

**Authors:** KS Joshi, VK Campbell, RJ Gooch, CM Anstey, MD Landy

**Affiliations:** Sunshine Coast Hospital and Health Service, Intensive Care Unit, Nambour, Australia

## Introduction

Hospital systems for the recognition (afferent limb) and management (efferent limb) of deteriorating patients, or Rapid Response Systems (RRS), are being mandated world-wide. This is despite conflicting evidence regarding their efficacy, with studies criticised for afferent limb failure, and questionable end points1. Additionally there are concerns regarding treatment limitation decisions and ward staff deskilling. Still, as elsewhere, we were admitting patients into the Intensive Care Unit (ICU) who we felt we should have been involved with earlier.

## Methods

Our retrospective, single-center, observational study compared equivalent 18-month periods before-and-after the revised RRS. Revisions included the implementation of the Q-ADDS observation form[2], sustained staff education and feedback, mandatory call activation, and primary treating team leadership or co-leadership. Primary end points included RRS activation volume, improved illness severity of unplanned ICU admissions from the ward, ICU LOS and ICU mortality. Secondary end points included unplanned ICU admissions, cardio-respiratory arrests and hospital mortality.

## Results

With the new RRS, APACHE II (21 to 17, p < 0.001), APACHE III (68 to 64, p = 0.011) and SAPS (38 to 35, p = 0.044) scores at ICU admission from the ward were reduced. Less patients were in the severest quartile of APACHE II (17.5 to 6.5, p < 0.001), APACHE III (15.5 to 7.5, p = 0.012) and SAPS (24 vs 14, p = 0.006). Accordingly, ICU LOS (4 Vs. 3 days, p = 0.02) was reduced and prolonged stay (>7 days) also trended down (27% to 19%, p = 0.055). Organ support initiation (p = 0.3) and ICU mortality (25/181 (13.7%) vs. 33/239 (13.8%), p = 0.93) were unchanged, although the number were small. The frequency of RRS activation (11 Vs. 48/1000 admissions, p < 0.001) was increased, with only a 0.7% failure to initiate a response where indicated. Hospital mortality, albeit multifactorial, trended lower (1/57 Vs. 1/64 admissions, p = 0.06). Unplanned ICU admissions and arrests remain unchanged.

## Conclusions

This revised RRS has improved illness severity at ICU admission, reduced ICU length of stay in our hospital. These specific outcomes may provide a reasonable measure, of RRS efficacy, even in smaller centres.
